# Development, characterization, and immunomodulatory evaluation of a liposomal DNA vaccine targeting *Schistosoma mansoni* IPSE/α-1

**DOI:** 10.3389/fvets.2026.1910156

**Published:** 2026-10-22

**Authors:** Danah A. Alturbak, Abdullah A. Alshehri, Fahad A. Almughem, Somayah J. Jarallah, Abdulaziz Alouffi, Mohammed A. Marie, Doaa M. Elnagar, Alanoud T. Aljasham

**Affiliations:** 1 Department of Clinical Laboratory Sciences, College of Applied Medical Sciences, King Saud University, Riyadh, Saudi Arabia; 2 Advanced Diagnostics and Therapeutics Institute, Health Sector, King Abdulaziz City for Science and Technology (KACST), Riyadh, Saudi Arabia; 3 Department of Knowledge and Institutional Excellence, King Abdulaziz City for Science and Technology (KACST), Riyadh, Saudi Arabia; 4 Department of Zoology, College of Science, King Saud University, Riyadh, Saudi Arabia

**Keywords:** cytotoxicity, DNA-vaccine, IPSE/alpha-1, liposomes, nanoparticles, pVAX-1, *Schistosoma mansoni*

## Abstract

**Background/objectives:**

*Schistosoma mansoni* remains a major public health problem in endemic areas and requires innovative vaccine strategies. This research aimed to develop a DNA vaccine targeting the immune-modulatory antigen Interleukin-4-inducing principle from *S. mansoni* eggs alpha-1 (IPSE/alpha-1), a major secretive glycoprotein from *S. mansoni* eggs known to induce IL-4 production and promote a Th2-type immune response.

**Methods:**

The IPSE/alpha-1 gene was codon-optimized for mammalian expression, cloned into the pVAX-1 vector, transformed into DH5α, and confirmed by colony PCR before plasmid extraction. The DNA vaccine was complexed in liposomal nanoparticles formulated with DC-Chol and DOPE lipids at a 1:1 molar ratio by thin-film hydration followed by manual extrusion. The formulations were prepared at N/P ratios (1:1 to 6:1) and characterized by particle size and zeta potential. Cytotoxicity was assessed using the MTS assay on Human Foreskin Fibroblast-1 (HFF-1) cells. *In vivo* experiments were conducted using 100 male albino mice allocated into four groups receiving PBS or 1, 2, or 3 μg/mL of the formulated vaccine to assess the modulation of pro- and anti-inflammatory cytokines.

**Results:**

The formulation with an N/P ratio of 3:1 yielded nanoparticles with a mean diameter of <200 nm and a zeta potential of approximately +30 mV. The MTS assay revealed high cell viability at concentrations ≤1 μg/mL, while higher concentrations showed dose-dependent cytotoxicity. The IC_50_ was estimated between 2–4 μg/mL. *In vivo* experiments showed dose- and time-dependent immune modulation. Across IFN-γ, IL-4, and IL-10, the 2 μg/mL dose consistently produced the strongest and most sustained responses. IFN-γ showed delayed but robust activation, IL-4 rose steadily from 48 h onward, and IL-10 displayed early elevation followed by regulated late increases.

**Conclusion:**

The results confirmed the successful construction and characterization of a liposome-complexed DNA vaccine targeting IPSE/alpha-1 with favorable physicochemical properties and low cytotoxicity at lower concentrations. *In vivo* cytokine analysis demonstrated dose- and time-dependent modulation of immune responses, highlighting the immunomodulatory potential of the developed formulation. These findings support future studies on immunogenicity and protective efficacy against *S. mansoni* infection.

## 1 Introduction

Schistosomiasis, commonly referred to as bilharzia, is an acute and chronic parasitic disease caused by trematodes of the genus *Schistosoma* and remains a major public-health concern in tropical and subtropical regions. According to the Global Burden of Disease 2021 estimates, schistosomiasis affected approximately 151.4 million people worldwide and accounted for nearly 1.75 million disability-adjusted life years (DALYs) and 12,858 deaths in 2021 (1). More recent WHO data indicate that 253.7 million people required preventive treatment in 2024 and that approximately 14,353 deaths are attributable to schistosomiasis annually, although mortality is likely underestimated (2).

*Schistosoma mansoni* is a major causative agent of intestinal schistosomiasis, which can lead to chronic abdominal, hepatic, and intestinal morbidity (3). Its life cycle involves a freshwater snail intermediate host and a human definitive host. Snail-derived cercariae penetrate human skin and develop into adult worms that reside in the mesenteric veins. Female worms produce eggs that are either excreted in feces to continue transmission or retained in intestinal and hepatic tissues, where they provoke granulomatous inflammation and fibrosis. The central role of retained eggs in disease pathology provides a biological rationale for investigating egg-secreted proteins involved in host–parasite interactions and immune modulation (4).

In Saudi Arabia, both *S. mansoni* and *Schistosoma haematobium* are endemic, with *S. mansoni* infections primarily reported in the highlands of the western regions, as well as in specific areas of the Central and Northern regions. Epidemiological studies have documented intestinal schistosomiasis in various parts of the country, highlighting the need for effective control (5, 6). Despite ongoing control programs, schistosomiasis persists in certain areas, underscoring the need for new preventive strategies, including vaccine development.

Current control strategies primarily rely on mass drug administration of praziquantel (7). However, this approach does not prevent reinfection, and concerns about drug resistance highlight the urgent need for effective vaccines (8). DNA vaccines have emerged as a promising avenue due to their ability to induce both humoral and cellular immune responses, stability, and ease of production (9).

Schistosomiasis principally induces complex immune responses characterized by dynamic shifts in cytokine profiles that shape both pathology and protection. Infection typically triggers an early Th1-type response, followed by a dominant Th2-mediated cytokine environment that drives granuloma formation and tissue remodeling (10). Modulation of these cytokine networks is central to host immunity and has become a key target in vaccine development. Among the antigens investigated, Sm14 is a 14-kDa fatty acid-binding protein of *S. mansoni* involved in fatty acid binding and intracellular transport (11–13). Recombinant Sm14 has demonstrated protective activity in experimental infection models, and its formulation with GLA-SE has shown favorable tolerability and antigen-specific immunogenicity in human clinical trials (14). These findings support its development as a vaccine candidate.

IPSE/α-1 is an abundant and immunologically active glycoprotein secreted by *S. mansoni* eggs that is involved in host–parasite interactions and egg-induced immune modulation. Its ability to induce basophil-derived IL-4 and promote Th2-associated responses makes it a biologically relevant target for investigating the effects of DNA-based antigen delivery. However, its intrinsic immunomodulatory activity may itself influence systemic cytokine profiles and should not be interpreted as evidence of protective or antigen-specific immunity (15, 16).

Although several vaccine candidates against *S. mansoni* have previously been investigated, including recombinant proteins, peptide-based vaccines, attenuated parasites, and nanoparticle-based delivery systems, limited studies have explored liposome-complexed DNA vaccines targeting the egg-secreted antigen IPSE/alpha-1. This antigen was selected to investigate its immunomodulatory potential following DNA-based delivery, rather than on the basis of demonstrated superiority to Sm14.

The present study describes the construction of a DNA vaccine candidate encoding the codon-optimized *IPSE/alpha-1* gene, its cloning into the pVAX-1 expression vector, and subsequent complexation in liposomal nanoparticles. The study aimed to characterize the formulation’s physicochemical properties, evaluate its *in vitro* cytotoxicity, and assess its *in vivo* effects on cytokine responses. Together, these analyses provide a foundational dataset on the formulation and its immunomodulatory potential to support subsequent studies of antigen-specific immunogenicity and protective efficacy.

## 2 Materials and methods

### 2.1 Optimization of the IPSE/alpha-1 sequence

The selection of the target gene *IPSE/alpha-1* from *S. mansoni* was based on previous studies that showed that IPSE/alpha-1 is a major immunogenic component secreted from *S. mansoni* eggs (15). The amino acid sequence of IPSE/alpha-1 is available in the UniProt database under the accession number Q869D4. To optimize gene expression in mammalian cells, the gene *IPSE/alpha-1* was designed to have specific codons that enhance the efficiency of translation, avoid rare codons, and reduce mRNA secondary structure that may disrupt expression. Moreover, two restriction enzyme sites, HindIII (AAGCTT) and KpnI (GGTACC), were introduced to the 5′ and 3′ ends, respectively, to facilitate cloning into the PVAX-1 vector, which has the same restriction enzyme sites. The optimized *IPSE/alpha-1* was synthesized by GenScript Biotech (Singapore).

### 2.2 Cloning, transformation, and purification of pVAX1–IPSE/α-1

The PVAX-1 plasmid and the 510 bp *IPSE/alpha-1* insert were digested with the restriction enzymes HindIII and KpnI (Jena Bioscience) at 37 °C for 2 h. The digested products were confirmed by agarose gel electrophoresis and purified using a gel extraction kit (ThermoScientific, United States). The purified *IPSE/alpha-1* insert was ligated into purified PVAX-1 plasmid using T4 DNA ligase enzyme (Thermo Scientific, United States) at a 3:1 inserts to vector ratio for 3 h at room temperature. Following ligation, the reaction mixture was purified using the Wizard® SV Gel and PCR Clean-Up System (Promega, United States) to remove residual ligation components and concentrate the ligation products. The ligation product was then transformed into chemically competent *E. coli* DH5α cells by heat shock transformation method. Transformants were selected on Luria-Bertani (LB) agar plates supplemented with 50 μg/mL kanamycin following overnight incubation at 37 °C. Five colonies were screened by colony PCR using primers Forward: 5′TAAGCAG AGCTCTCTGGCTAAC3′, and Reverse: 5′TTGGTGAAGTCGT CGATGCAG3′. PCR was carried out with an initial denaturation at 95 °C for 3 min, followed by 34 cycles consisting of denaturation at 95 °C for 30 s, annealing at 58 °C for 30 s, and extension at 72 °C for 1 min. A final extension at 72 °C for 5 min was included. The PCR Positive colonies were sub-cultured into LB broth containing 50 μg of kanamycin and incubated overnight at 37 °C. Next day, the plasmids were extracted using QIAGEN Plasmid Plus Maxi Kit (Qiagen, Netherlands) and their concentrations were measured using a NanoDrop spectrophotometer (Thermofisher, United States).

### 2.3 Liposomal nanoparticle preparation and physicochemical characterization

The liposome-complexed DNA vaccine targeting *S. mansoni* (IPSE/alpha-1) was prepared using the thin-film hydration technique followed by manual extrusion. The cationic lipid 3β- [N-(N′, N′-dimethyl aminoethane)-carbamoyl] cholesterol hydrochloride (DC-Chol) (MW: 537.6 g/mol) and the helper lipid 1,2-dioleoyl-sn-glycero-3-phosphoethanolamine (DOPE) (MW: 744.02 g/mol) were used at a molar ratio of 1:1. For each formulation, 12.5 μg of DNA was used, and six formulations were prepared, plus an additional 5% to account for handling losses, totaling 78.75 μg. The DNA solution was prepared by diluting 24.6 μL of concentrated DNA stock (3.201 μg/μL) in nuclease-free water to a final volume of 6.3 mL. To investigate the impact of cationic charge on complex formation, a range of nitrogen-to-phosphate (N/P) ratios—1:1, 2:1, 3:1, 4:1, 5:1, and 6:1—was prepared, representing the molar ratio between the positively charged amine groups of DC-Chol and the negatively charged phosphate groups of the DNA.

DC-Chol and DOPE were dissolved separately in a chloroform: methanol mixture (5:1, v/v). Both solutions were then combined in a round-bottom flask, and the organic solvents were evaporated under nitrogen gas to form a uniform thin lipid film. The film was then hydrated with the prepared DNA solution. After hydration, the mixture was vortexed vigorously to disperse the lipid film and form multi-lamellar vesicles. The manual extrusion was then performed using a 200 nm polycarbonate membrane filter mounted between two syringes. The lipid dispersion was passed through the membrane 21 times at 50 °C to facilitate uniform vesicle formation. The resulting liposomes were collected, labeled, and stored at 4 °C for immediate use or −80 °C for long-term storage.

### 2.4 Particle size, zeta potential, and complexation analysis

Particle size distribution and hydrodynamic diameter were determined using dynamic light scattering (DLS) analysis with a Zetasizer Nano ZS system. Measurements were conducted after completion of the extrusion process using a 200 nm polycarbonate membrane. Although extrusion through 200 nm membranes may commonly produce particles with slightly larger hydrodynamic diameters, repeated extrusion cycles (21 passes) performed at 50 °C contributed to improved vesicle homogeneity and reduced size variability. The optimized formulation demonstrated a narrow particle size distribution with mean diameters below 200 nm, supporting its suitability for enhanced cellular uptake and nanoparticle-mediated gene delivery applications (17). DNA complexation analysis was qualitatively assessed using 1% agarose gel electrophoresis prepared in 1 × TAE buffer and stained with ethidium bromide (EtBr). Electrophoresis was run at 100 V for 45 min. The degree of DNA complexation was inferred by comparing the intensity of DNA bands from free and liposome-bound samples. The absence or retardation of DNA migration indicated successful complexation. All physicochemical characterization experiments, including particle size and zeta potential measurements, were performed in triplicate to ensure reproducibility and statistical reliability. Data are presented as mean ± standard deviation (SD).

### 2.5 *In vitro* cytotoxicity assessment by MTS assay

The *in vitro* cytotoxicity of the liposome-complexed DNA vaccine formulation was assessed using the MTS assay according to (18). The assay was conducted on HFF-1 cells (human foreskin fibroblast cells) (SCRS-1041), maintained in Dulbecco’s Modified Eagle Medium (DMEM; Gibco, United States) supplemented with 10% fetal bovine serum (FBS), 100 U/mL penicillin, and 100 μg/mL streptomycin. Cells were seeded at 1.5 × 10^4^ cells per well in a 96-well flat in 100 μL of complete DMEM. After overnight incubation to allow adherence, the culture medium was replaced with 100 μL of serum-free DMEM containing increasing concentrations of the liposomal formulation (ranging from 0.125 to 16 μg/mL). Wells containing medium alone served as the positive control, while those treated with 0.1% Triton X-100 served as the negative control. All treatments were performed in triplicate and incubated for 24 h at 37 °C in a 5% CO_2_ incubator.

Following incubation, the media was discarded, and 120 μL of MTS reagent mixture (20 μL MTS + 100 μL DMEM) was added to each well. Plates were covered in aluminum foil and kept for 3-4 h. Absorbance was then measured at 490 nm using a Cytation 3 microplate reader (BioTek Instruments Inc., United States). Cell viability (%) was calculated using the formula:


$$\text{Cell Viability}\%=\frac{S-T}{H-T}\times 100.$$

here, *S* denotes the absorbance of cells treated with the test formulations, *T* represents the absorbance of cells exposed to Triton X-100, and *H* corresponds to the absorbance of cells maintained in DMEM. Reported values are expressed as the mean ± SD from at least three independent experiments.

### 2.6 *In vivo* analysis of cytokine modulation induced by synthesized schistosomula vaccine in animal model

One hundred healthy male Swiss albino mice (25–30 g, 14 weeks old) were obtained from the Central Laboratory, King Saud University. Animals were housed under standard laboratory conditions (22 ± 2 °C, 12 h light/dark cycle, relative humidity 50–60%) with free access to a standard pellet diet and water provided ad libitum. Mice were acclimatized for 1 week prior to the start of the experiment. All experimental procedures were conducted in accordance with institutional guidelines for the care and use of laboratory animals.

### 2.7 Experimental design

One hundred male mice were randomly distributed into four main groups (n = 25 per group): the first group received phosphate-buffered saline (PBS). The second group received the anti-schistosomal vaccine at 1 μg/mL. The third group received the anti-schistosomal vaccine at 2 μg/mL. The fourth group received the anti-schistosomal vaccine at 3 μg/mL. Each main group was further subdivided into five subgroups (*n* = 5 per subgroup) according to the time of sacrifice for sample collection: Subgroup A: Animals were sacrificed 24 h post-vaccination. Subgroup B: Animals were sacrificed 48 h post-vaccination. Subgroup C: Animals were sacrificed 7 days post-vaccination. Subgroup D: Animals were sacrificed 14 days post-vaccination. Subgroup E: Animals were sacrificed 21 days post-vaccination. At each time point, mice were euthanized using CO_2_ flow, and blood samples were collected for cytokine measurement of IFN-γ, IL-4, and IL-10 to assess systemic cytokine responses following formulation administration (Figure 1).

**Figure 1:**
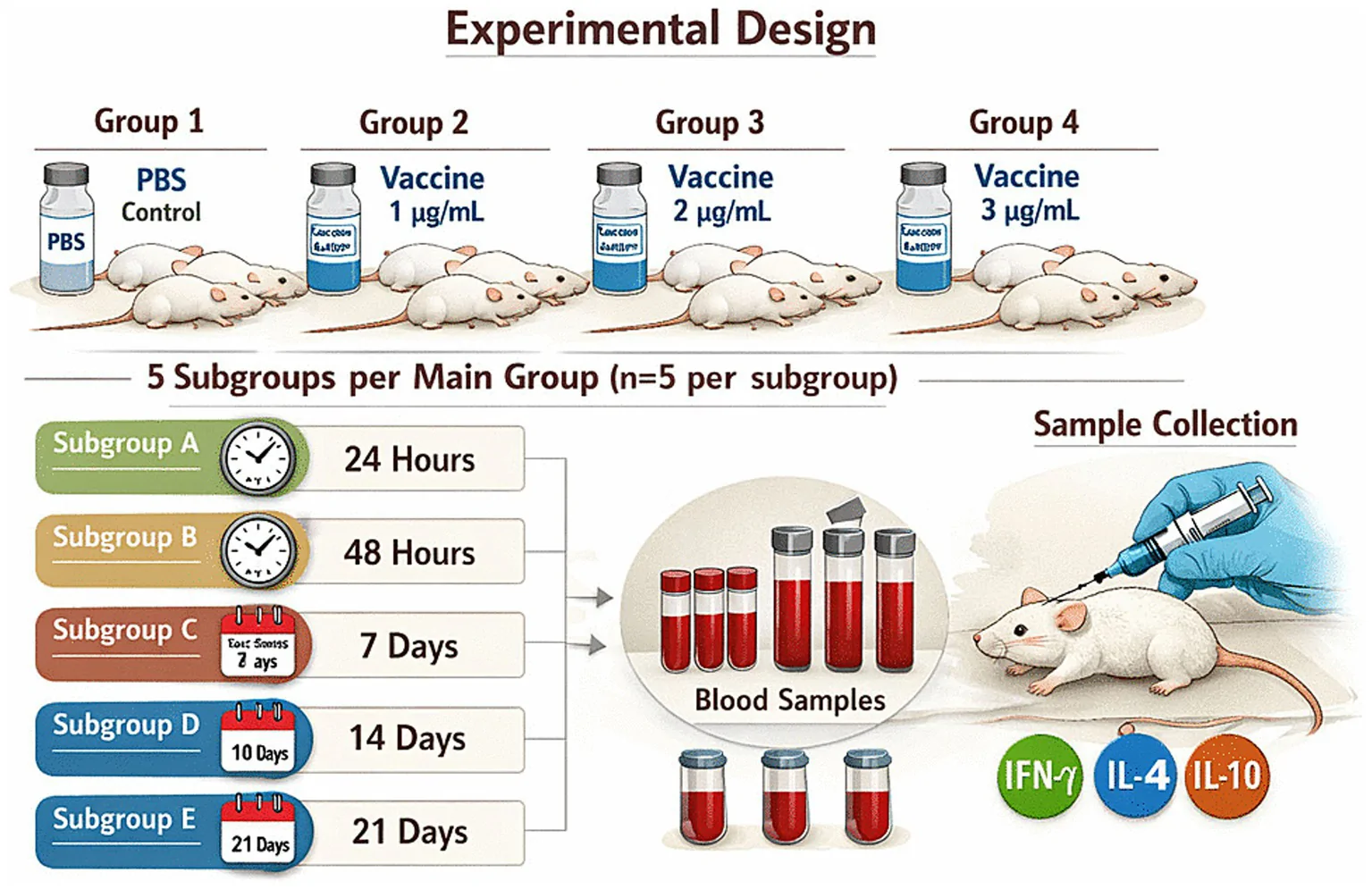
Schematic diagram of experimental design.

### 2.8 Determination of proinflammatory IFN- γ and IL-4 and anti-inflammatory cytokine IL-10

All reagents are allowed to reach room temperature (20–25 °C) before the assay is initiated. A volume of 200 μL Wash Buffer is added to each well. The plate was sealed and mixed on a plate shaker for 10 min at room temperature (20–25 °C). The Wash Buffer was decanted, and residual liquid was removed by inverting the plate and tapping it firmly onto absorbent towels. A volume of 25 μL of each Standard or Control was added to the designated wells. Assay Buffer was used for the 0 pg./mL background standard. A volume of 25 μL Assay Buffer was added to all sample wells. A volume of 25 μL of the appropriate matrix solution was added to the background, standard, and control wells. A volume of 25 μL of each diluted serum sample was added to the appropriate wells. The mixing bottle was vortexed, and 25 μL of mixed was added to each well.

The plate was sealed, wrapped in foil, and incubated with agitation on a plate shaker overnight (16–18 h) at 2–8 °C for optimal sensitivity, precision, and accuracy. Well contents were gently removed, and the plate was washed twice according to the plate washing instructions. A volume of 25 μL of detection antibodies—Anti-Mouse IFNγ (MIFNG-MAG), Anti-Mouse IL-4 (MIL4-MAG), Anti-Mouse IL-10 (MIL10-MAG)—pre-warmed to room temperature was added to each well. The plate was sealed, covered with foil, and incubated with agitation for 1 h at room temperature (20–25 °C); the wells were not aspirated after this incubation. A volume of 25 μL Streptavidin-Phycoerythrin was added to each well. The plate was sealed, covered with foil, and incubated with agitation for 30 min at room temperature (20–25 °C). Well contents were gently removed, and the plate was washed twice according to the plate washing instructions. A volume of 150 μL Drive Fluid PLUS for MAGPIX® was added to all wells, and the beads are re-suspended on a plate shaker for 5 min. The plate was run on a Luminex® 200™ Bio-Plex system instrument using the appropriate software. Median Fluorescent Intensity (MFI) data are saved and analyzed using a 5-parameter logistic or spline curve-fitting method to calculate cytokine concentrations.

### 2.9 Statistical analysis

All experiments were performed in triplicate unless otherwise stated, and data are presented as mean ± standard deviation (SD) or mean ± standard error of the mean (SEM), as indicated in the corresponding figure legends. Statistical analyses were performed using GraphPad Prism version 10 (GraphPad Software, United States). Differences between experimental groups were analyzed using one-way analysis of variance (ANOVA) followed by Tukey’s *post hoc* multiple comparison test. For all analyses, a *p*-value <0.05 was considered statistically significant. Statistical significance was represented as follows*: p* < 0.05 (*), *p* < 0.01 (**), *p* < 0.001 (***), and *p* < 0.0001 (****), while ns indicates non-significant differences.

## 3 Results

### 3.1 Optimization and synthesis of the IPSE/alpha-1 gene

The IPSE/alpha-1 sequence from *S. mansoni* was selected as the target antigen based on its immunogenic properties. To ensure high expression efficiency in mammalian systems, the gene was codon-optimized and synthesized with flanking HindIII and KpnI restriction sites for directional cloning into the pVAX-1 expression vector. The optimized sequence incorporated mammalian-preferred codons and eliminated rare codons and secondary mRNA structures that could inhibit translation (Supplementary Figure S1).

### 3.2 Cloning, transformation, and purification of pVAX1–IPSE/α-1

The synthesized IPSE/alpha-1 gene was digested with HindIII and KpnI and successfully ligated into the similarly digested pVAX-1 plasmid. The ligation mixture was incubated with T4 DNA ligase, and the recombinant plasmid was then transformed into chemically competent *E. coli* DH5α cells. Colonies were selected on LB-kanamycin plates and screened by colony PCR using pVAX-specific primers. Amplification of the expected product confirmed successful cloning of the IPSE/alpha-1 insert into the pVAX-1 vector (Figure 2). The purified plasmids were quantified by NanoDrop spectrophotometry, yielding high-concentration DNA suitable for downstream applications, including nanoparticle complexation and animal immunization.

**Figure 2:**
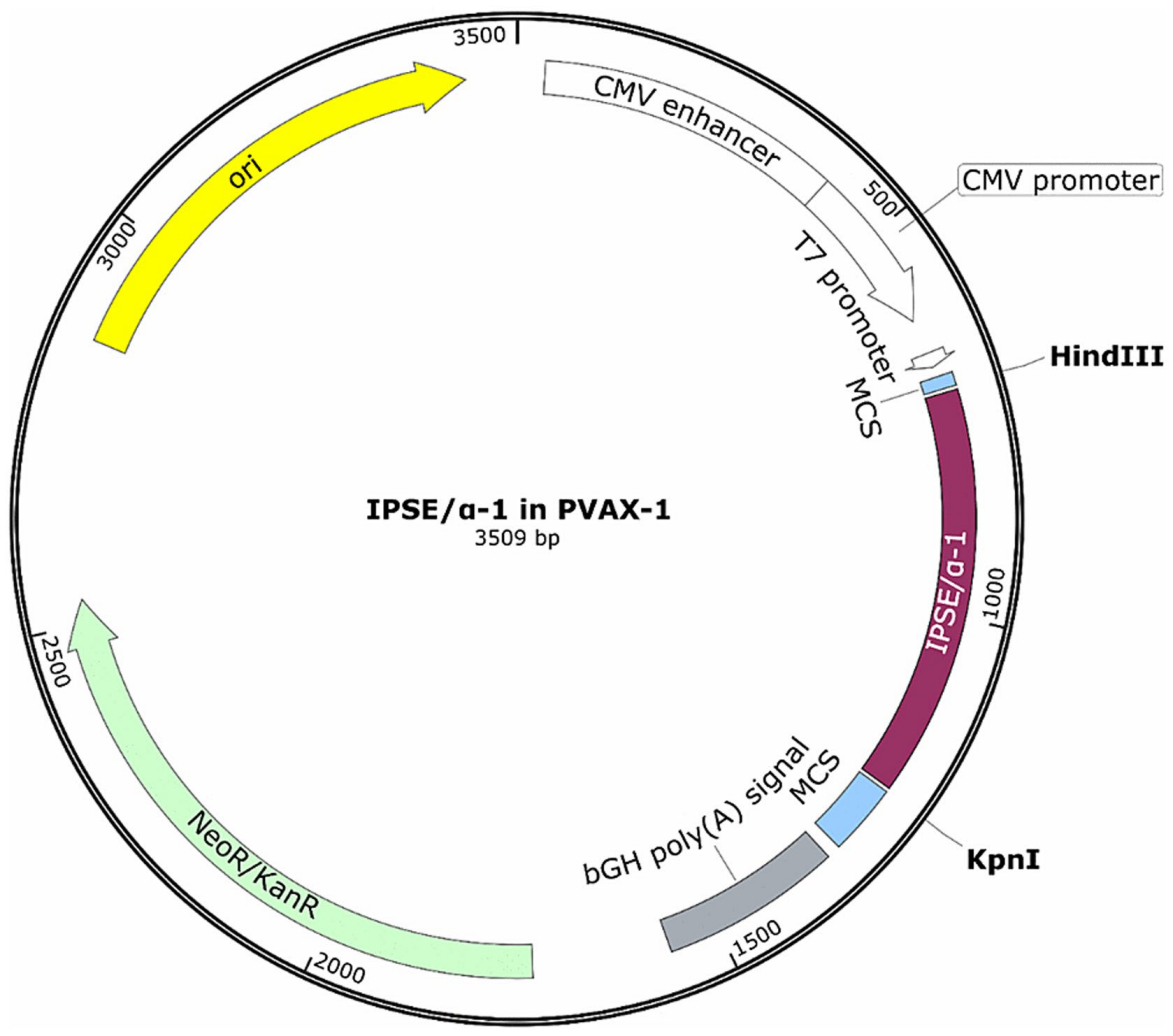
Schematic representation of the optimized *IPSE/alpha-1* sequence cloned into the pVAX-1 plasmid and flanked by HindIII and KpnI restriction sites. This figure was created by SnapGene 8.1.1.

### 3.3 Preparation and physicochemical characterization of liposome- complexed DNA vaccine

The DNA vaccine was complexed into liposomes using the thin-film hydration method, followed by manual extrusion. The DC-Chol: DOPE was used at 1:1 molar ratio. Following solvent evaporation and hydration with plasmid DNA solution, the formulation underwent extrusion through a 200 nm polycarbonate filter to achieve uniform particle size.

Dynamic light scattering analysis revealed a relatively homogeneous nanoparticle population with low size dispersion following repeated extrusion. The positive zeta potential (~+30 mV) indicated successful electrostatic interaction between the cationic lipids and plasmid DNA, which may contribute to nanoparticle stability and improved cellular internalization efficiency (19, 20). The optimized 3:1 formulation demonstrated stable physicochemical characteristics with low variability between replicates, confirming the reproducibility of the formulation process and nanoparticle stability during analysis.

### 3.4 Gel-retardation assay

The complexation of the DNA vaccine within the liposomal nanoparticles was evaluated using an agarose gel-retardation assay. This method is based on the principle that free plasmid DNA migrates through an agarose gel under an electric field, while DNA complexed with nanoparticles shows reduced or no migration due to increased size and charge interactions, indicating successful complexation. As shown in Figure 3, the assay was performed using plasmid DNA (pVAX-IPSE/alpha-1) complexed with liposomes at various N/P charge ratios ranging from 1:1 to 6:1. The complete or partial retardation of DNA migration in Lanes 2 to 7 is indicative of successful complex formation. Gel-retardation analysis demonstrated increasing plasmid complexation with increasing N/P ratio. Residual migrating plasmid DNA was observed at N/P ratios of 1:1 and 2:1, whereas no detectable free DNA migration was observed at an N/P ratio of 3:1 or higher. Accordingly, 3:1 was identified as the minimum tested N/P ratio that achieved complete apparent plasmid retardation under the experimental conditions and was therefore selected as the optimal formulation for downstream biological assays. This qualitative retardation indicates electrostatic association between the plasmid DNA and the liposomal formulation; however, DNA protection, cellular uptake, and transfection efficiency were not directly evaluated (Figure 3). Densitometric analysis of the agarose gel-retardation assay was performed using Fiji/ImageJ, with naked plasmid as the 100% free-DNA reference, to estimate the fraction of plasmid associated with the liposomes. This analysis strengthens formulation characterization, and the results are provided in Supplementary Figure S2. Although the gel densitometry provided quantitative evidence of efficient plasmid complexation, absolute encapsulation efficiency was not independently determined by separation of free and nanoparticle-associated DNA. Therefore, the present data should be interpreted as demonstrating efficient plasmid association and electrophoretic retention rather than direct measurement of the direct liposomal DNA encapsulation. Naked plasmid bands had an integrated intensity of 9,803.47 arbitrary units; the 1:1 formulation showed 791.79 units of migrated DNA, equivalent to approximately 8.1% free DNA and 91.9% apparent complexation/retardation. At ratios of 1:2 and higher, we detected no clear plasmid bands in the naked-plasmid migration region; DNA fluorescence remained at or near the loading region, consistent with complete or near-complete complexation under the assay’s detection conditions.

**Figure 3:**
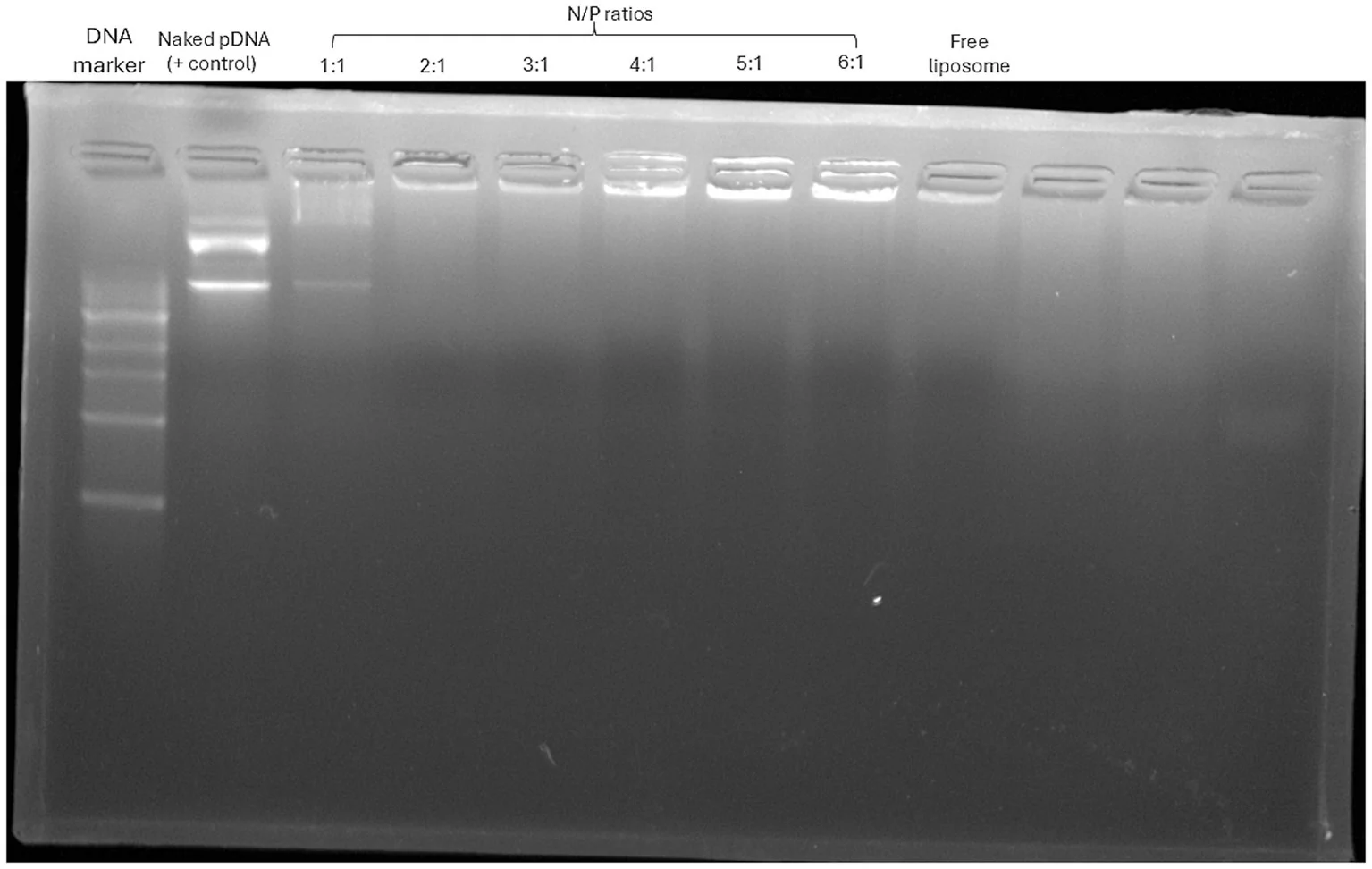
Relative metabolic activity of HFF-1 cells treated with increasing concentrations of nanoparticle- complexed DNA vaccine as measured by the MTS assay after 24 h of exposure. Cells were incubated with formulations ranging from 0.125 to 16 μg/mL. Cell viability was expressed as a percentage relative to untreated controls (positive control: media only), with Triton X-100 (0.1%) used as the cytotoxic reference (negative control). Each bar represents the mean ± standard deviation (*n* = 3). A dose-dependent decline in metabolic activity was observed, with minimal cytotoxicity at ≤1 μg/mL and significant reduction in viability at concentrations ≥2 μg/mL.

### 3.5 Cytotoxicity evaluation of DNA-loaded nanoparticles using MTS assay

To assess the biocompatibility of the nanoparticle-formulated DNA vaccine, an MTS cytotoxicity assay was conducted on HFF-1 cells following 24-h treatment at increasing concentrations ranging from 0.125 to 16 μg/mL. The relative metabolic activity, expressed as a percentage of the untreated control as shown in (Figure 4).

**Figure 4:**
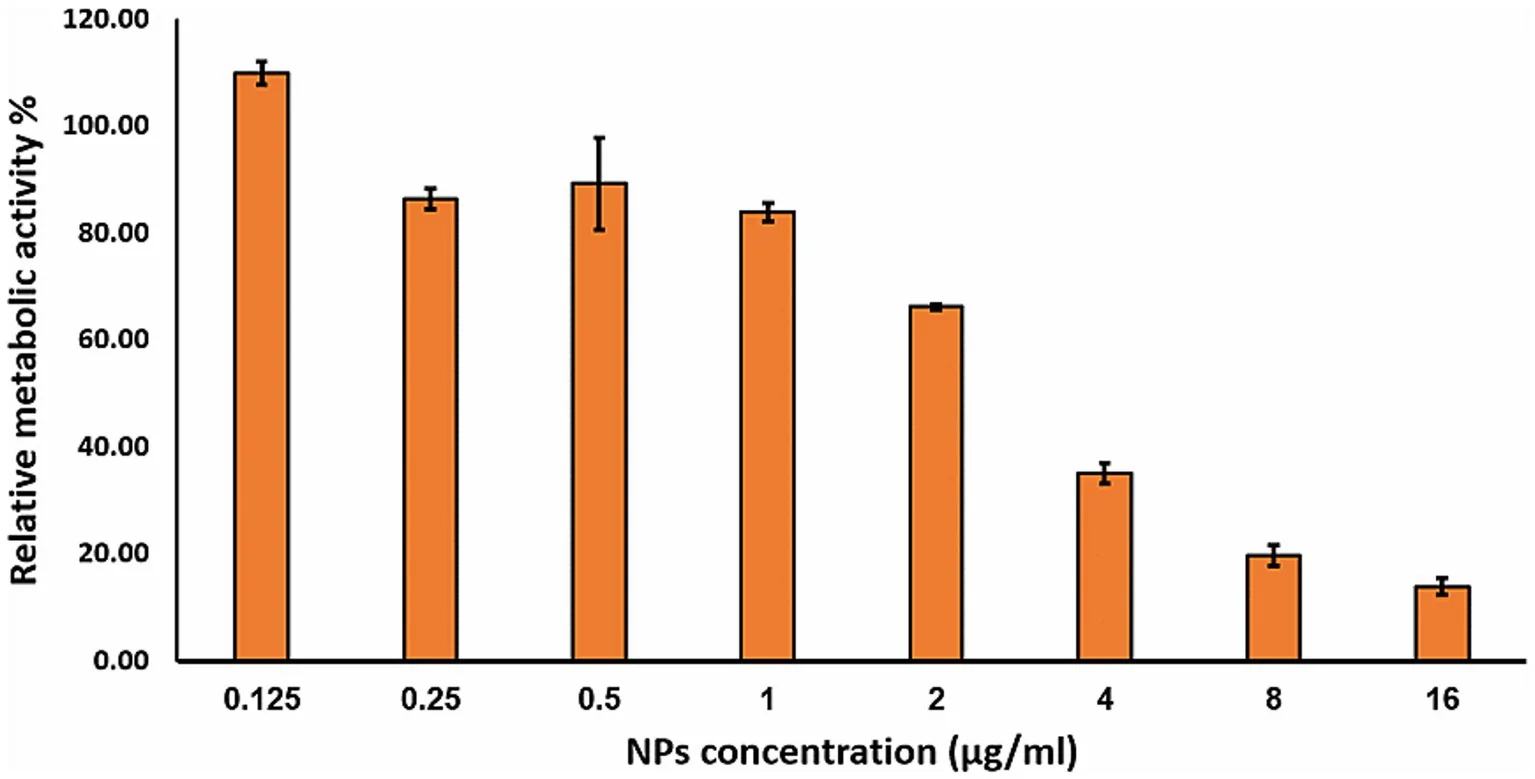
Agarose gel-retardation assay demonstrating qualitative DNA complexation analysis of liposomal formulations with varying N/P ratios at a fixed DC-Chol: DOPE molar ratio of 1:1. Lane 1: Naked pVAX-IPSE/alpha-1 plasmid (positive control); Lanes 2–7: liposome: DNA complexes with increasing N/P ratios of 1:1, 2:1, 3:1, 4:1, 5:1 and 6:1, respectively; Lane 8: free liposome. Complete or partial retardation of DNA in lanes 2–7 indicates successful complexation.

Cell viability remained high at lower concentrations. At 0.125 μg/mL, metabolic activity reached 109.82% ± 2.14, indicating no cytotoxicity and a possible proliferation-promoting effect. Mild reduction was observed at 0.25, 0.5 μg/mL, maintaining >85% viability. However, at 2 μg/mL and above, a dose-dependent decrease was noted, with viability dropping to 66.20%, 35.06%, 19.79%, and 13.92% for 2, 4, 8, and 16 μg/mL, respectively.

These results demonstrate that DNA-loaded nanoparticles exhibit low cytotoxicity at concentrations ≤1 μg/mL, with significantly reduced metabolic activity at higher doses. The IC_50_ appears to fall between 2–4 μg/mL, indicating the upper safety limit for *in vitro* applications.

### 3.6 Evaluation of dose-dependent IFN-γ responses following anti-schistosomal vaccination in mice

IFN-γ levels showed dose- and time-dependent variation following administration of the formulations. During the early phase (24–48 h), IFN-γ levels were lower in all treated groups than in the control group. By day 7, IFN-γ remained reduced in the 1 and 3 μg/mL groups, whereas partial recovery was observed in the 2 μg/mL group. At day 14, IFN-γ levels increased markedly, particularly in the 1 and 2 μg/mL groups. This increase persisted through day 21, when the 2 μg/mL group exhibited the highest IFN-γ level among the tested groups. These findings demonstrate temporal and dose-dependent changes in systemic IFN-γ levels; however, they do not establish antigen-specific cellular immunity or Th1 polarization (Supplementary Figure S3).

The temporal patterns of IFN-γ secretion reveal a clear interaction between vaccine dose and the kinetics of cellular immune activation. At 1 μg/mL, IFN-γ production rises rapidly, reaching its maximum at 48 h before declining steadily through days 7–21, indicating a strong but short-lived early response. In contrast, the 2 μg/mL dose elicits a more gradual increase, culminating in a pronounced peak at day 14, consistent with the maturation of adaptive immunity and suggesting this concentration most effectively sustains T-cell activation over time. The 3 μg/mL dose produces uniformly low IFN-γ levels across all time points, with no significant fluctuations, implying either immune suppression at higher antigen loads or insufficient stimulation of IFN-γ–producing cells. Together, these patterns demonstrate that intermediate dosing optimizes IFN-γ–mediated immunity, while both lower and higher concentrations generate weaker or poorly sustained responses (Figure 5).

**Figure 5:**
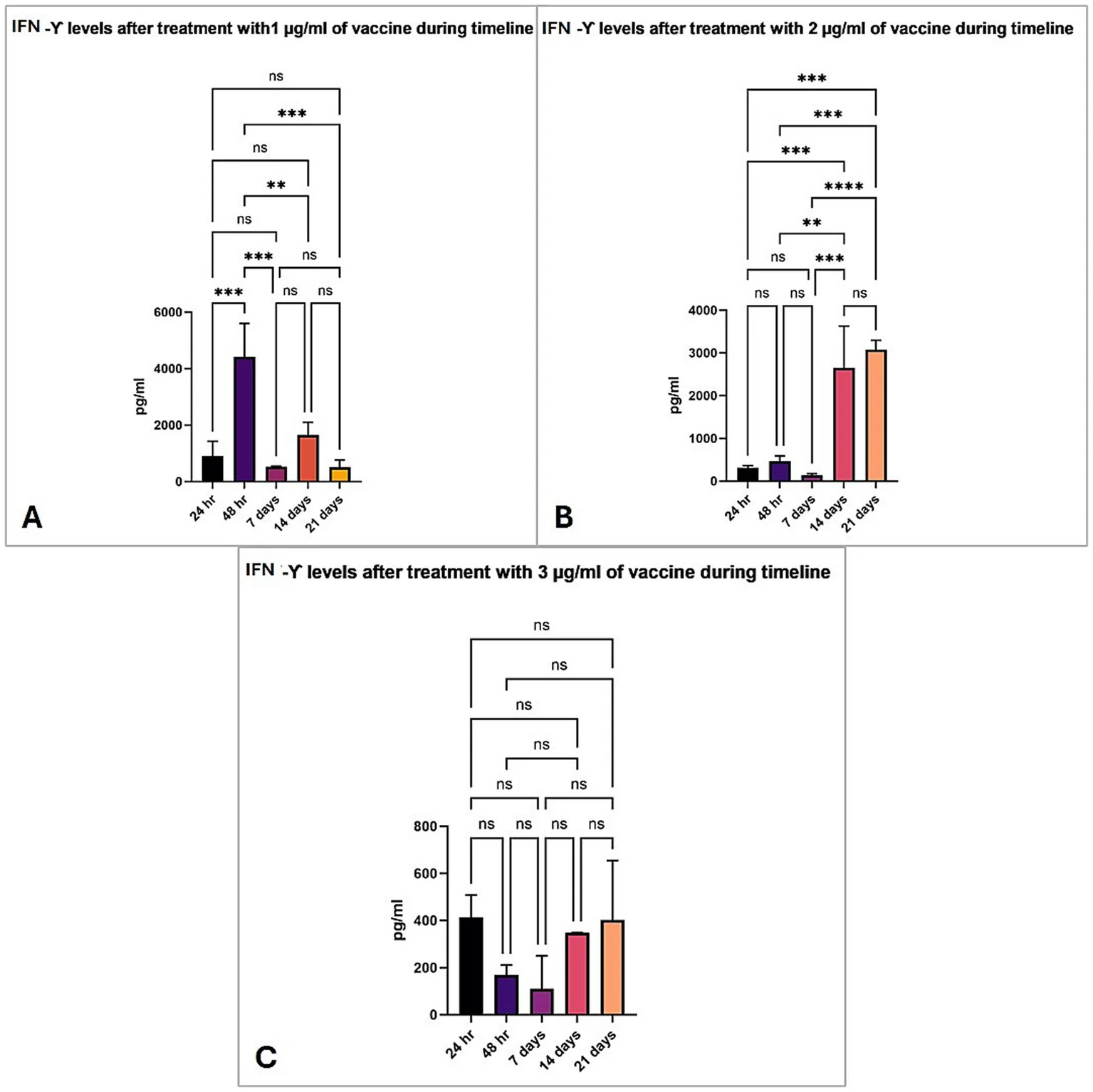
Interferon-γ (IFN-γ) production over time following exposure to increasing vaccine concentrations. Bar graphs show IFN-γ levels (pg/ml) measured at 24 h, 48 h, 7 days, 14 days, and 21 days after treatment with 1 μg/mL **(A)**, 2 μg/mL **(B)**, or 3 μg/mL **(C)** of the vaccine. Each bar represents mean IFN-γ concentration ± standard deviation. The 1 μg/mL dose produced an early peak at 48 h, followed by a decline at later time points. The 2 μg/mL dose showed a delayed but robust peak at 14 days, indicating strong activation of cellular immunity. The 3 μg/mL dose resulted in consistently low IFN-γ levels across all time points, with no statistically significant changes. Statistical comparisons between time points are indicated as follows: ns, not significant; *p < 0.05; **p < 0.01; ***p < 0.001; ****p < 0.0001. Analyses were performed using GraphPad Prism version 10.

### 3.7 Assessment of dose-dependent IL-4 responses following anti-schistosomal vaccination in mice

The present findings demonstrated a time- and dose-dependent modulation of IL-4 production following administration of the anti-schistosomal vaccine. At 24 h post-vaccination, no significant differences were observed among groups, indicating that early IL-4 response was minimal. However, a marked elevation in IL-4 levels was detected at 48 h, particularly in the 2 μg/mL group, suggesting an acute inflammatory activation at this intermediate dose. This elevated response persisted through days 7, 14, and 21, where the 2 μg/mL dose consistently induced the highest IL-4 concentrations compared with control and other vaccinated groups. Interestingly, the 3 μg/mL dose did not further enhance IL-4 production, implying a possible dose-threshold effect or regulatory feedback mechanism limiting excessive cytokine release. These findings indicate that the 2 μg/mL formulation produced the highest systemic IL-4 response during the later time points against schistosomal infection (Supplementary Figure S4).

The temporal analysis of IL-4 production following administration of different doses of the anti-schistosomal vaccine revealed a dynamic and dose-dependent cytokine response. In the 1 μg/mL group, IL-4 levels showed a moderate increase at 48 h and peaked around day 7, followed by a gradual decline at days 14 and 21, indicating a transient inflammatory activation. In contrast, the 2 μg/mL dose induced a more robust and sustained elevation of IL-4, with a significant rise beginning at 48 h and remaining markedly elevated through days 7, 14, and 21, suggesting prolonged immune stimulation at this concentration. The 3 μg/mL group exhibited an early increase at 48 h, with fluctuating but sustained levels up to day 21, although the magnitude of response was lower than that observed with the 2 μg/mL dose. Collectively, these findings indicate that IL-4 production is both time- and dose-dependent, with the intermediate dose (2 μg/mL) eliciting the strongest and most sustained pro-inflammatory response, which may reflect an optimal balance between immune activation and regulatory control (Figure 6).

**Figure 6:**
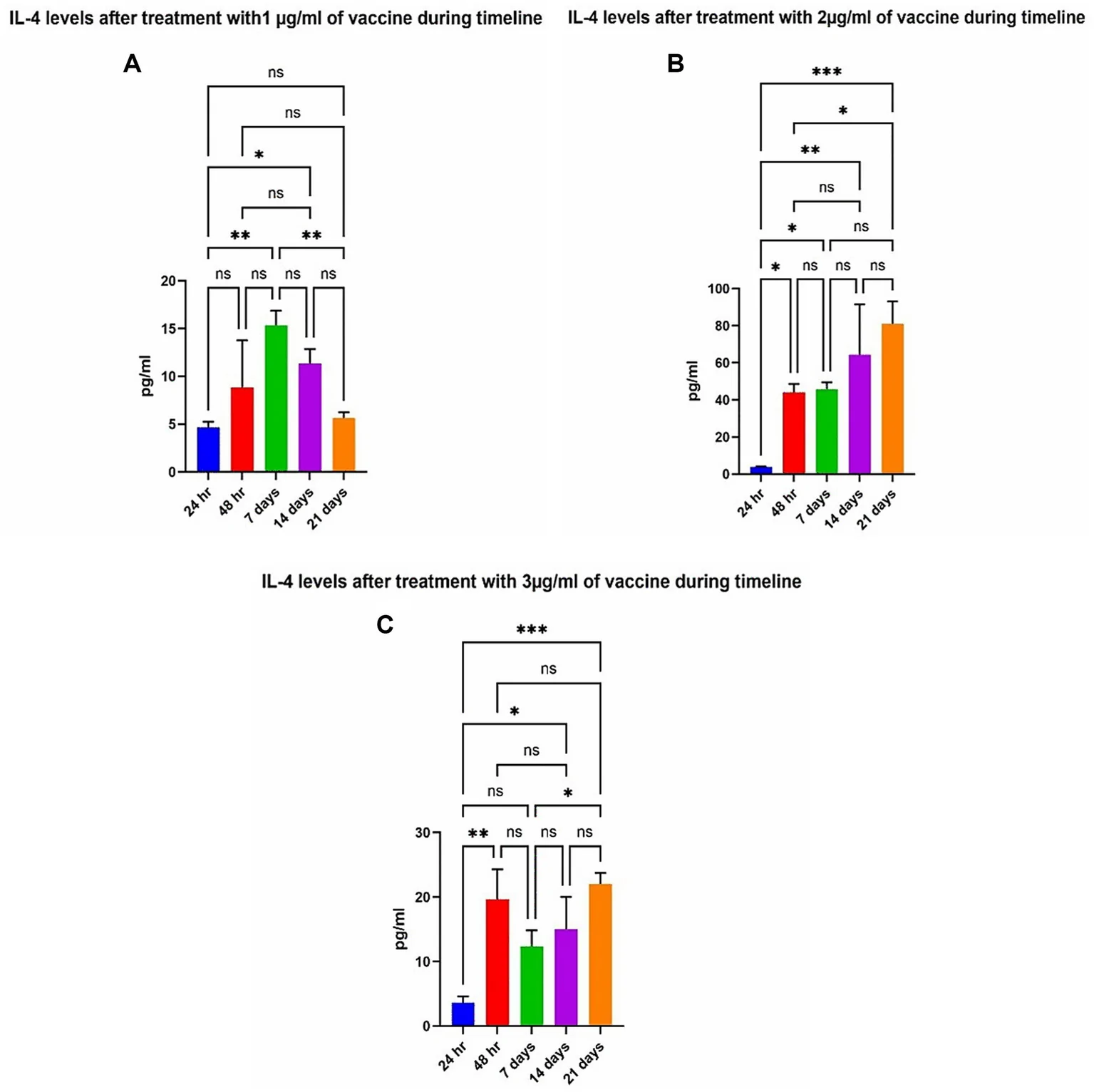
Temporal changes in serum IL-4 levels following administration of different doses of anti-schistosomal vaccine. Serum interleukin-4 (IL-4) concentrations (pg/mL) were measured at 24 h, 48 h, 7 days, 14 days, and 21 days after vaccination with **(A)** 1 μg/mL, **(B)** 2 μg/mL, and **(C)** 3 μg/mL of the anti-schistosomal vaccine. Data are expressed as mean ± SEM. Statistical analysis was performed using one-way ANOVA followed by a *post hoc* multiple comparison test to evaluate differences between time points within each dose group. Statistical comparisons between time points are indicated as follows: ns, not significant; *p < 0.05; **p < 0.01; ***p < 0.001; ****p < 0.0001. Analyses were performed using GraphPad Prism version 10.

### 3.8 Dose-dependent modulation of IL-10 responses following anti-schistosomal vaccination in mice

IL-10 showed a clear time- and dose-dependent pattern after vaccination. Levels rose sharply within the first 24 h, especially in the 3 μg/mL and 2 μg/mL groups, while the 1 μg/mL dose produced only a mild increase. At 48 h, IL-10 continued to rise, with the highest values again in the 3 μg/mL group, indicating an early regulatory immune response. By day 7, IL-10 declined across all groups, and differences were no longer significant, suggesting a temporary reduction in anti-inflammatory activity. At day 14, IL-10 increased again, peaking in the 2 μg/mL group, whereas the 3 μg/mL group showed a notable drop compared with earlier time points. By day 21, IL-10 remained elevated in the 2 μg/mL group and moderately increased in the 3 μg/mL group. Overall, the vaccine produced a dynamic, dose-responsive modulation of IL-10, reflecting a regulated and evolving immune response over the course of immunization. Overall, these findings indicate that the anti-schistosomal vaccine induced a time- and dose-dependent modulation of IL-10, reflecting the development of a regulated immune response following vaccination (Supplementary Figure S5).

The IL-10 response showed distinct dose- and time-dependent patterns across the three vaccine concentrations. In the 1 μg/mL group, IL-10 fluctuated mildly, dipping at 48 h and 7 days before peaking at 14 days and then returning to earlier levels by day 21. The 2 μg/mL group displayed an early rise at 24 h, followed by a decline through 48 h and 7 days, but then showed a strong rebound at 14 days that remained elevated at 21 days, indicating a delayed but sustained regulatory response. The 3 μg/mL group produced very high IL-10 levels during the early phase (24–48 h), followed by a sharp drop at 7 days and persistently low levels at 14 days, with a moderate increase again by day 21 that did not reach the initial peak. Overall, IL-10 dynamics varied markedly by dose, reflecting different regulatory immune trajectories induced by each vaccine concentration.

In summary, these findings indicate that IL-10 production following vaccination exhibits dose-dependent and time-dependent variations, with higher vaccine doses inducing a stronger early regulatory response, while intermediate doses appear to sustain IL-10 production during later stages of the immune response (Figure 7).

**Figure 7:**
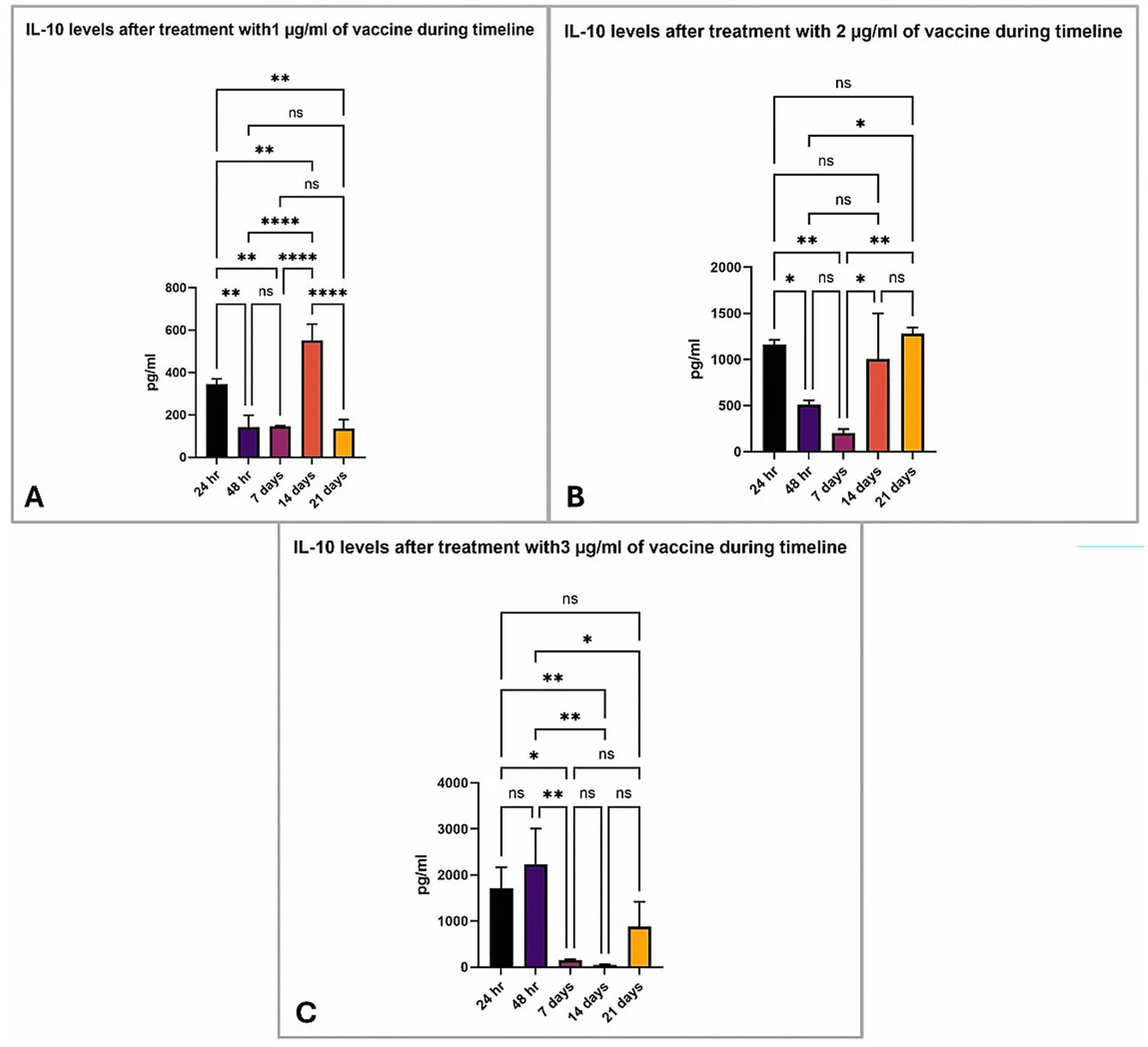
Temporal changes in IL-10 levels following treatment with different concentrations of the anti-schistosomal vaccine. Serum IL-10 levels were monitored at 24 h, 48 h, 7 days, 14 days, and 21 days following administration of 1 **(A)**, 2 **(B)**, or 3 **(C)** μg/mL of the anti-schistosomal vaccine. **(A)** The 1 μg/mL group showed mild fluctuations with a peak at day 14. **(B)** The 2 μg/mL group exhibited an early rise at 24 h, a decline through 48 h and 7 days, and a marked increase at 14 and 21 days. **(C)** The 3 μg/mL group displayed high IL-10 levels during the early phase (24–48 h), followed by a sharp decrease at 7 and 14 days, with partial recovery by day 21. Data are expressed as mean ± standard deviation (SD). Statistical analysis was performed using one-way ANOVA followed by a post-hoc multiple comparison test to assess differences among time points within each treatment group. Statistical comparisons between time points are indicated as follows: ns, not significant; *p < 0.05; **p < 0.01; ***p < 0.001; ****p < 0.0001. Analyses were performed using GraphPad Prism version 10.

## 4 Discussion

This study successfully demonstrates the construction, formulation, *in vitro* and *in vivo* evaluation of a liposome-complexed DNA vaccine targeting the immunomodulatory IPSE/alpha-1 antigen of *S. mansoni*. Strategic codon optimization of IPSE/alpha-1 for mammalian expression and directional cloning into the pVAX-1 vector provides a plasmid platform for mammalian expression; however, transgene expression was not experimentally confirmed in the present study. The use of pVAX-1 is supported by its safety profile and successful application in other DNA vaccines (21–23).

Our work is grounded in the immuno-biological relevance of IPSE/alpha-1, a glycoprotein secreted from *S. mansoni* eggs known to modulate host immunity by inducing IL-4 release from basophils and supporting a Th2-polarized immune response (16). Prior studies have confirmed IPSE/alpha-1’s capacity to downregulate inflammation in murine models of allergic airway disease, positioning it as a dual-use candidate for both vaccine and immunotherapy applications (24). The *IPSE/alpha-1* gene cloned in this study retains the full-length, active sequence, and its incorporation into a plasmid vaccine platform represents a novel translational step in vaccine design against schistosomiasis.

Complexation of the DNA into liposomes using DC-Chol and DOPE lipids produced nanoparticles under 200 nm with a zeta potential of approximately +30 mV, properties consistent with effective cellular uptake and endosomal escape (25). These findings align with previous research on DC-Chol-based lipoplexes for gene delivery, which showed enhanced transfection efficiency and prolonged antigen presentation *in vivo* (20, 26), the DC-Chol:DOPE molar ratio of 1:1, combined with an N/P ratio of 3:1, was optimal in balancing DNA complexation, particle size, and surface charge, corroborating earlier observations on cationic lipid-based vaccine delivery (27).

Compared with previously reported schistosomiasis vaccine candidates, the present formulation introduces several distinctive features, including the use of a codon-optimized IPSE/alpha-1 DNA construct combined with a DC-Chol/DOPE liposomal delivery platform. Previous studies have primarily focused on recombinant protein vaccines, peptide antigens, or adjuvant-based formulations, whereas comparatively fewer investigations have evaluated liposome-mediated DNA vaccine delivery targeting IPSE/alpha-1. In addition, the present study integrates physicochemical characterization, *in vitro* cytotoxicity analysis, and *in vivo* cytokine modulation within a single experimental framework, providing a broader evaluation of vaccine performance and biological activity. These findings support the potential applicability of liposomal DNA vaccines as promising platforms for schistosomiasis immunotherapy and prevention.

Our cytotoxicity results further validate the safety profile of the formulation. The MTS assay showed no adverse effects on cell viability at concentrations up to 1 μg/mL, consistent with previous findings on low-toxicity liposome-DNA complexes (28). A dose-dependent decline in viability at higher concentrations emphasizes the need for careful dose selection before *in vivo* evaluation. The estimated IC_50_ of 2–4 μg/mL characterizes cytotoxicity under the tested *in vitro* conditions (29); further studies are required to establish *in vivo* safety.

This work contributes to a growing body of literature emphasizing the promise of liposome-mediated DNA vaccines against parasitic infections. To our knowledge, this is the first study to combine codon-optimized IPSE/α-1, pVAX-1, and DC-Chol-based liposomal delivery. Future studies should focus on assessing immunogenicity in murine models, evaluating Th2 cytokine profiles, and testing protective efficacy against *S. mansoni* challenge.

A dynamic, dose- and time-dependent pattern of IFN-γ regulation emerged following vaccination, reflecting the cytokine’s central role in coordinating Th1-mediated immunity during schistosomiasis. IFN-γ was markedly suppressed across all vaccine doses during the early phase (24–48 h), suggesting an acute regulatory response that transiently dampens pro-inflammatory signaling—an effect consistent with early innate immune modulation observed in helminth vaccine models. By day 7, IFN-γ remained low in the 1 and 3 μg/mL groups, whereas the 2 μg/mL dose showed partial recovery, indicating that antigen load influences the timing and magnitude of Th1 reactivation. A pronounced shift occurred by day 14, when IFN-γ levels rose sharply, particularly at 1 and 2 μg/mL, signifying the onset of adaptive cellular immunity and the expansion of Th1 effector responses. This heightened activation persisted through day 21, with the 2 μg/mL dose eliciting the strongest IFN-γ production, suggesting that intermediate antigen concentrations may optimally stimulate Th1 polarization while avoiding the suppressive or dysregulated responses associated with suboptimal or excessive dosing. These findings align with previous studies demonstrating that IFN-γ is essential for protective immunity against schistosomes, driving macrophage activation, granuloma modulation, and parasite control, while its expression is tightly regulated by antigen dose and immune context (30, 31).

IL-4 showed a clear time- and dose-dependent pattern after vaccination. No meaningful changes appeared at 24 h, indicating that the earliest immune phase did not strongly activate IL-4. By 48 h, IL-4 rose sharply—especially in the 2 μg/mL group—suggesting that this intermediate dose effectively triggered early Th2-associated inflammatory signaling. This heightened IL-4 response continued through days 7, 14, and 21, where the 2 μg/mL dose consistently produced the strongest cytokine levels. The lack of further IL-4 increase at 3 μg/mL suggests a dose-threshold or regulatory feedback mechanism that prevents excessive cytokine release. Overall, the pattern indicates that the 2 μg/mL formulation produced the highest IL-4 levels; however, the cellular source and antigen specificity of this response remain undetermined (31, 32). Humoral immunity is relevant to schistosomiasis, although its contribution to protection depends on antigen specificity and antibody isotype. In human *S. mansoni* infection, IgE responses against adult-worm antigens have been associated with resistance to reinfection (33); however, this association cannot be directly extended to antibodies against the egg-secreted antigen IPSE/α-1. IPSE/α-1 can also induce IgE-dependent, antigen-independent IL-4 release from basophils (16). Thus, the elevated serum IL-4 observed in this study does not establish antigen-specific Th2 polarization or humoral immunity. Future studies should quantify IPSE/α-1-specific total IgG, relevant murine IgG subclasses, and IgE using validated ELISA methods before and after immunization. These measurements, combined with antigen-specific cellular assays and parasite challenge experiments, would help determine whether antibody responses are associated with reduced worm burden, tissue egg deposition, or pathology.

Vaccination against schistosomiasis induced a time-dependent modulation of Interleukin-10 (IL-10), indicating the activation of a regulatory immune response that varied according to vaccine dose and time after immunization. IL-10 levels increased markedly during the early phase (24–48 h), particularly at higher vaccine doses, suggesting an early immuno-regulatory mechanism that may control excessive inflammatory responses triggered by antigen exposure. IL-10 has been reported to limit inflammatory reactions during schistosome infection by suppressing excessive cytokine production and reducing immune-mediated tissue damage (34). At 7 days post-vaccination, IL-10 levels declined with no significant differences among groups, which may reflect a temporal change in systemic immune regulation; however, the present measurements cannot establish the underlying cellular mechanism (35). Interestingly, IL-10 levels increased again at later time points (14 and 21 days), particularly at intermediate vaccine doses, suggesting the establishment of immune regulation during the adaptive phase. IL-10 plays an important role in maintaining the balance between Th1 and Th2 responses, preventing excessive immune polarization and limiting immunopathology during schistosomiasis (36). Additionally, IL-10 has been shown to regulate parasite-specific T-cell responses and modulate lymphocyte activation during schistosome infection, which may explain the sustained IL-10 levels observed at later stages following vaccination (34–38). The concurrent elevation of IFN-γ, IL-4, and IL-10 indicates a mixed systemic cytokine response involving Th1-, Th2-, and regulatory-associated signals. IL-10 may limit excessive inflammatory activity; however, because the cellular sources of these cytokines were not determined, the present findings cannot establish the involvement of specific regulatory T-cell populations. It also remains unclear whether this cytokine profile would promote protection or limit vaccine efficacy. Antigen-specific cellular assays, phenotypic characterization, and challenge studies are needed to determine its biological significance.

A limitation of this study is that the final pVAX1-IPSE/α-1 construct was not independently verified by Sanger sequencing, and its expression in mammalian cells was not experimentally confirmed. Future studies should verify the insert and cloning junctions by sequencing and confirm transgene expression using RT-qPCR and protein-based methods. Furthermore, immunological assessment was limited to serum cytokine measurements, which indicate systemic immune modulation but do not establish IPSE/α-1-specific immunogenicity. Therefore, these findings should be considered preliminary, and future studies should evaluate antigen-specific humoral and cellular responses using appropriate assays.

## 5 Conclusion

This study developed and physicochemically characterized a liposome-complexed DNA vaccine candidate targeting the immunomodulatory IPSE/alpha-1 antigen of *S. mansoni*. Through codon optimization and cloning into the pVAX-1 vector, followed by complexation into DC-Chol/DOPE-based liposomes, the DNA vaccine candidate demonstrated favorable physicochemical properties, including nanoscale particle size and positive zeta potential. Importantly, the formulation exhibited qualitative plasmid DNA complexation in the gel-retardation assay and minimal cytotoxicity at lower concentrations. Although the gel-retardation assay confirmed plasmid DNA complexation qualitatively, future studies incorporating validated quantitative methods would enable a more precise determination of DNA loading capacity and complexation efficiency. The observed cytokine profiles suggest that the formulation may modulate systemic immune responses; however, they do not conclusively demonstrate IPSE/α-1-specific immunogenicity. Further studies evaluating antigen-specific humoral and cellular responses and protective efficacy are required to confirm the potential of this vaccine candidate for schistosomiasis control.

## Data Availability

The original contributions presented in the study are included in the article and its Supplementary material. Further inquiries can be directed to the corresponding authors.
