# Supplementary material for: Development, characterization, and immunomodulatory evaluation of a liposomal DNA vaccine targeting Schistosoma mansoni IPSE/α-1
Source: Front Vet Sci. 2026 Oct 22;13:1910156. doi: 10.3389/fvets.2026.1910156 (PMC13761767; doi:10.3389/fvets.2026.1910156)
Supplement: Supplementary file 1 [file Data_Sheet_1.PDF]

HindIII Start codon **IPSE/alpha-1** Stop codon KPnI

AAGCTTATGAACTTTCTGCTGTCTTGGGTGCATTGGAGCCTTGCCTTGCTGCTCTAC
CTCCACCATGCCAAGTGGTCCCAGGCTGCACCCATGGCAGAAGGAGGAGGGCAG
AATCATCACCATCATCACCATCACCATGGCGGAGAGAACCTGTATTTTCAGGGCGG
ATCCAACCCCTGCAAGTACTGCCTGCGGCTGTACGACGGCACCTACGAGAACGGC
AGCTACACCGACGTGTACAAGAGCGTGGGCAGCCTGAGCCCCCCCTGGATTCCTG
GCAGCGTGTGCGTGCCCCTGATCCACGACAGCAAGCGGCAGCCCCCCTACTGGC
GCCTGTACGAGGACGTGAACTACAGCGGCAAGGACACCGCCATCGGCCACGGCG
CCTGCATCGACGACTTCACCAAGAGCGGCCTGAAGCGGATCAGCAGCATCCAGA
AATGCGTGTACGGCGAGAATGGCATGGTGCAGTGCATCAGCGAGAGCAAGCGGC
GGAGAAAGTACTGCCGGTAC TAGGGTACC

Supplementary Figure S1: Codon-optimized Interleukin-4-inducing principle from *Schistosoma mansoni* eggs alpha-1 (*IPSE/alpha-1*) gene sequence showing the flanking HindIII (magenta) and KpnI (gray) restriction sites, start codon (ATG) (green), coding sequence (yellow), and stop codon (TAG) (red), designed for directional cloning into the pVAX-1 vector.

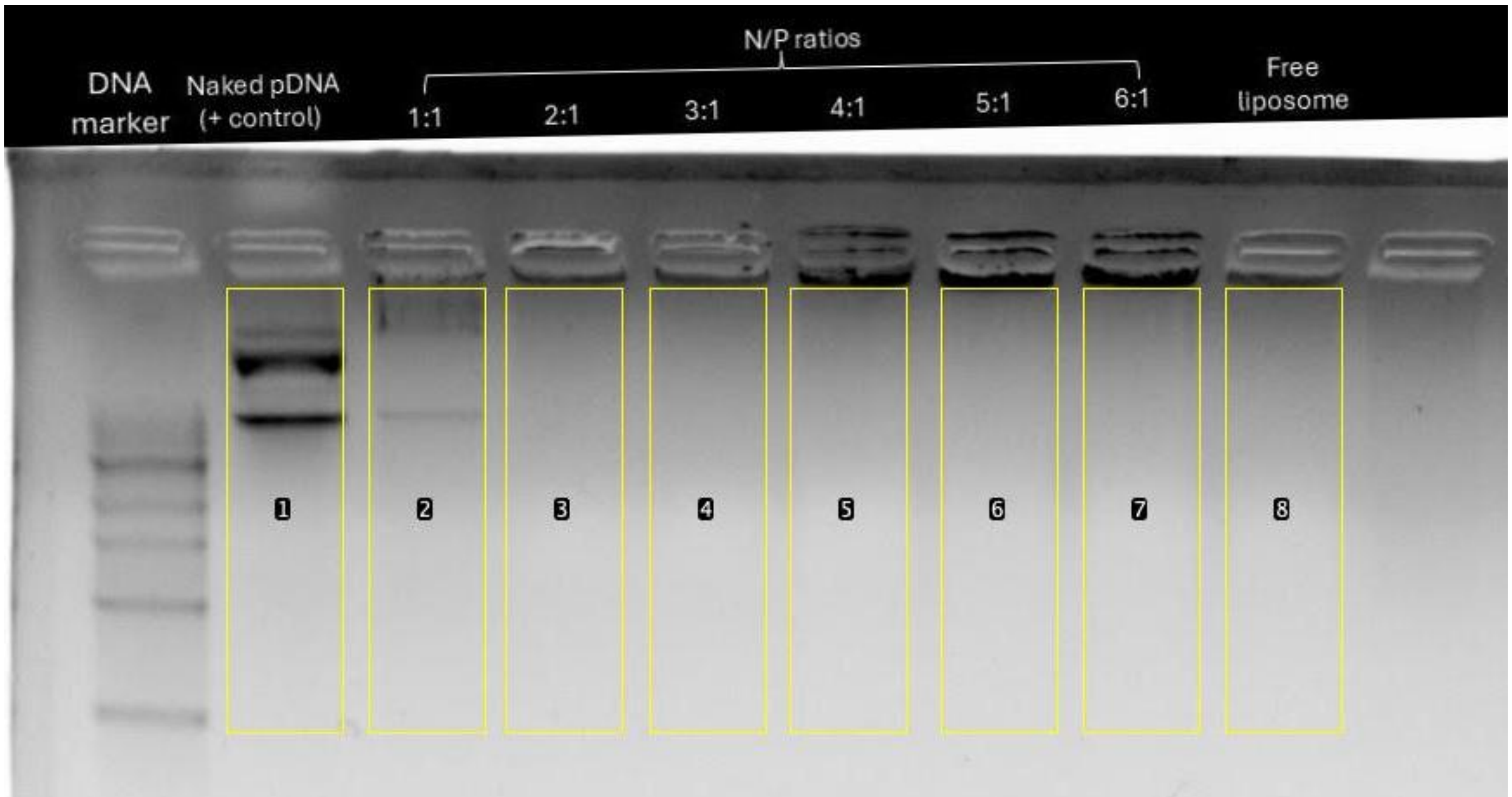

Supplementary Figure S2: Densitometric analysis of plasmid retardation in the agarose gel assay. Electrophoretically mobile DNA was quantified using Fiji/ImageJ and normalized to naked plasmid (100% free DNA; integrated intensity: 9,803.47 arbitrary units). At a 1:1 ratio, the migrated DNA intensity was 791.79 arbitrary units, corresponding to approximately 8.1% free DNA and 91.9% apparent plasmid association/retardation. At ratios of 2:1 or higher, no clear plasmid bands were detected in the naked-plasmid migration region, while fluorescence remained at or near the loading region. These results indicate efficient plasmid association and electrophoretic retention within the assay's detection limits and do not represent an absolute measure of intraliposomal encapsulation efficiency. Fiji (ImageJ)-image analysis software used for gel-band densitometry.

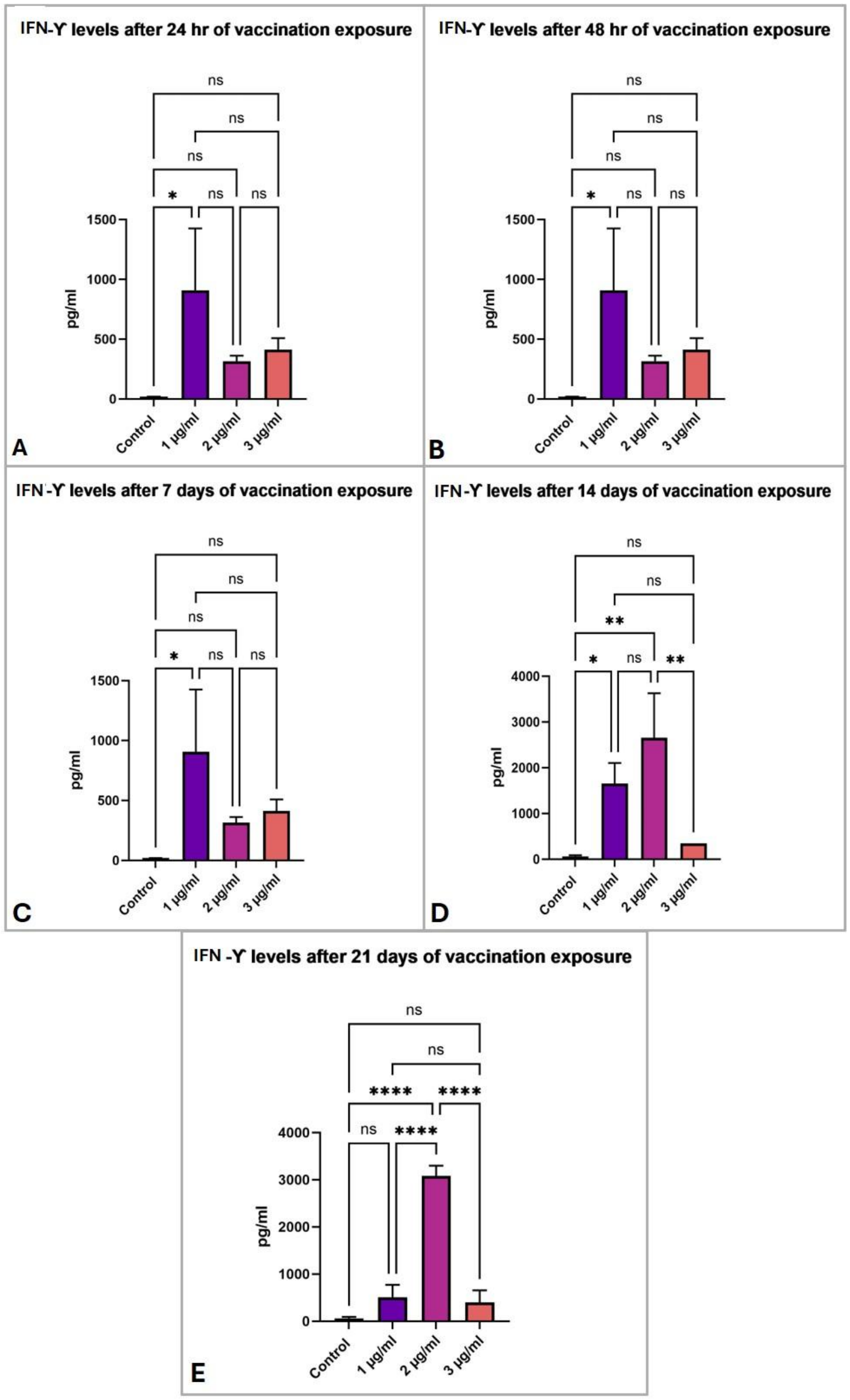

Supplementary Figure S3: IFN-γ levels in response to different concentrations of anti-schistosomal vaccine over time. IFN-γ levels (pg/ml) were measured in experimental groups of mice exposed to 1, 2, or 3 μg/mL of vaccine at different time points post-vaccination: (A) 24 hours, (B) 48 hours, (C) 7 days, (D) 14 days, and (E) 21 days. Data are presented as mean ± standard error of the mean (SEM). Statistical significance was assessed using one-way ANOVA followed by Tukey's multiple comparisons test. Statistical comparisons between time points are indicated as follows: ns, not significant; *$p < 0.05$; **$p < 0.01$; ***$p < 0.001$; ****$p < 0.0001$. Analyses were performed using GraphP ad Prism version 10.Notably, 1 μg/mL and 2 μg/mL doses induced significant increases in IFN-γ levels at later time points compared to control, whereas the 3 μg/mL dose showed reduced or non-significant responses at certain time points.

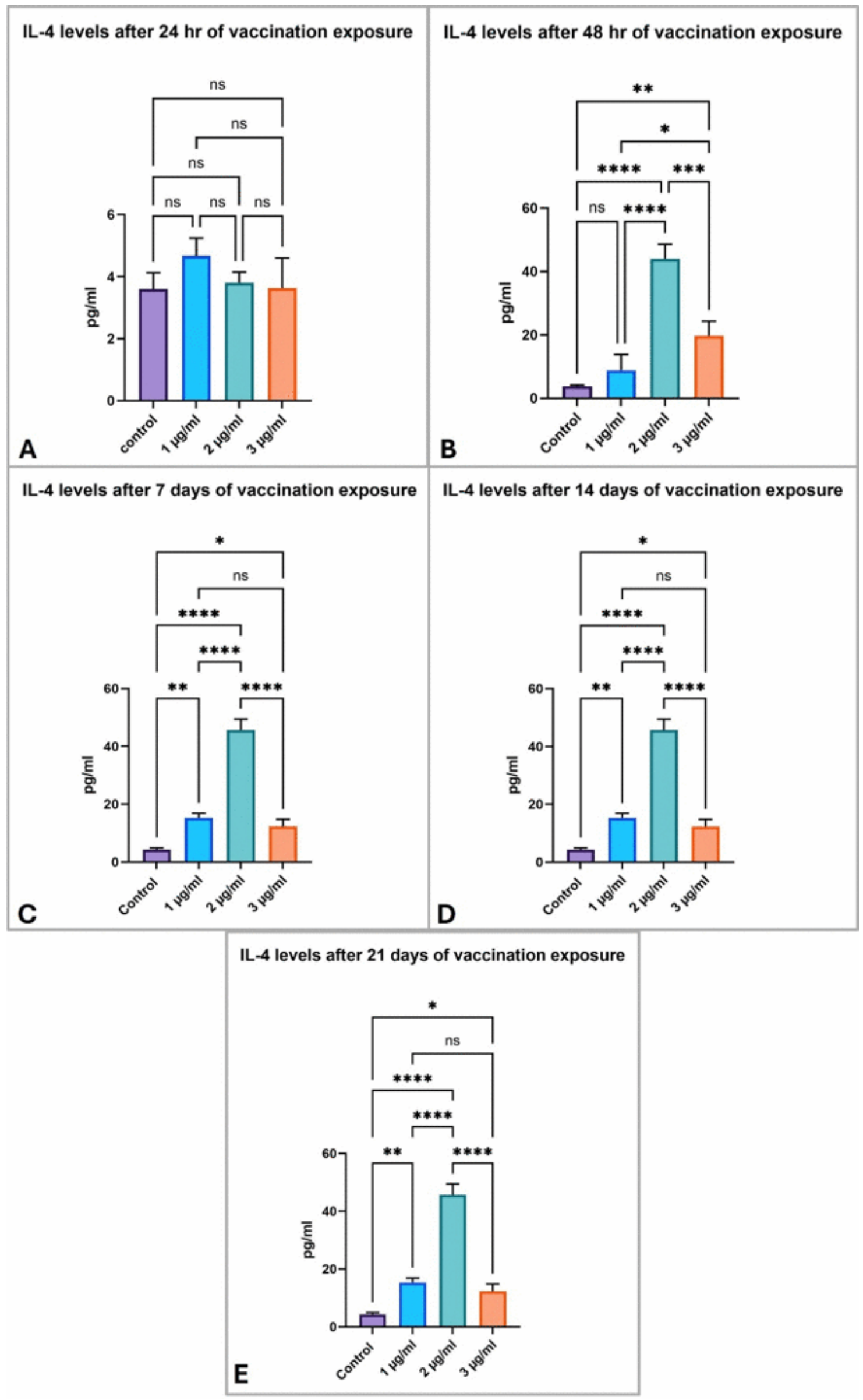

Supplementary Figure S4: Effect of different doses of anti-schistosomal vaccine on serum IL-4 levels at different time intervals.

Serum interleukin-4 (IL-4) levels (pg/mL) were measured in mice following administration of phosphate-buffered saline (Control) or anti-schistosomal vaccine at doses of 1, 2, and 3 µg/mL. IL-4 concentrations were assessed at (A) 24 h, (B) 48 h, (C) 7 days, (D) 14 days, and (E) 21 days post-vaccination. Data are presented as mean ± SEM. Statistical analysis was performed using one-way ANOVA followed by post hoc multiple comparison test. Statistical comparisons between time points are indicated as follows: ns, not significant; *$p < 0.05$; **$p < 0.01$; ***$p < 0.001$; ****$p < 0.0001$. Analyses were performed using GraphPad Prism version 10.

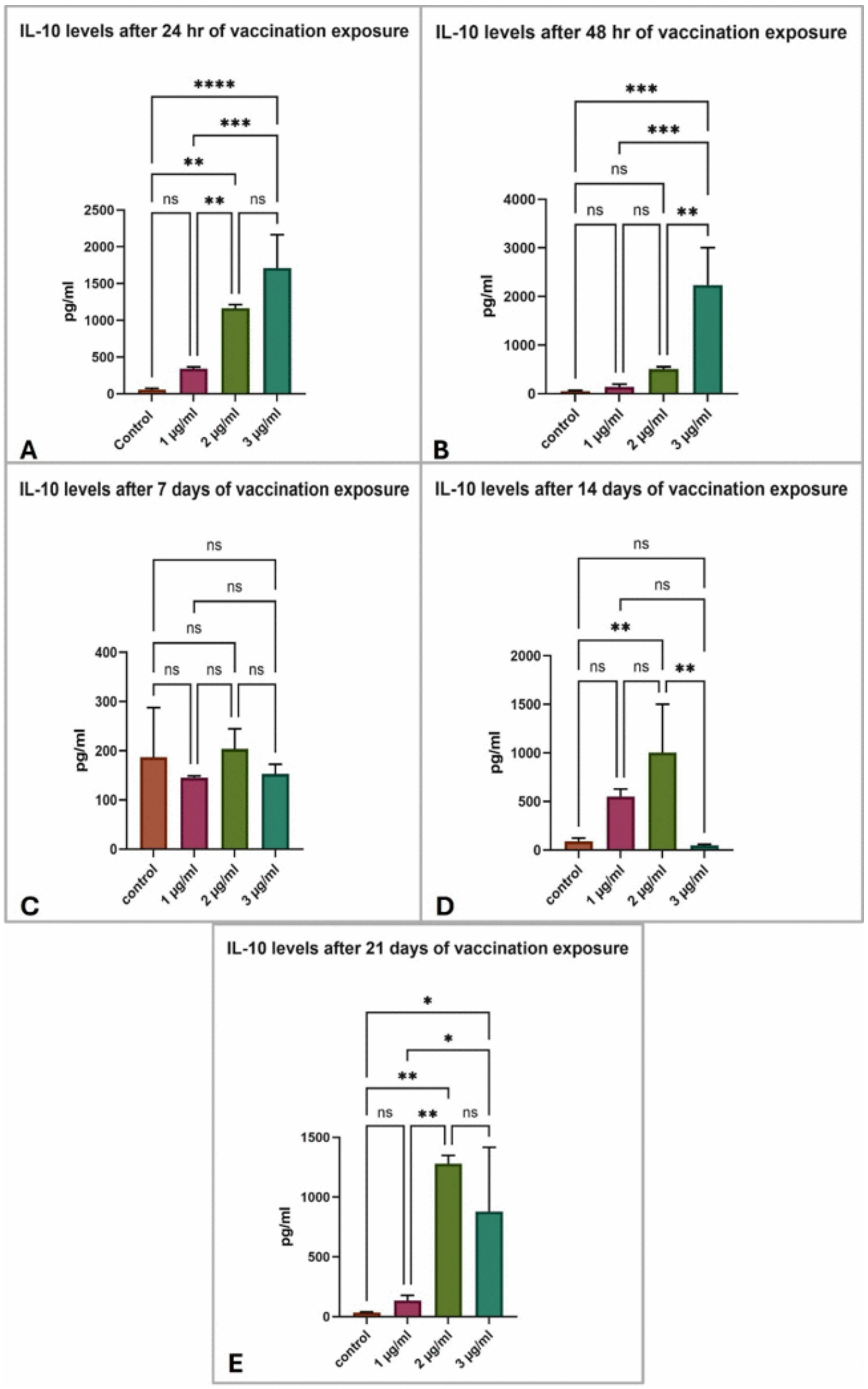

Supplementary Figure S5: Dose- and time-dependent changes in IL-10 levels following anti-schistosomal vaccination.

Serum IL-10 levels were measured in mice receiving PBS (control) or 1, 2, or 3 µg/mL of the anti-schistosomal vaccine at 24 h, 48 h, 7 days, 14 days, and 21 days post-vaccination. IL-10 increased in a dose-dependent manner at 24 h and 48 h, with the 3 µg/mL group showing the highest levels. By day 7, IL-10 levels were similar across groups. At day 14, IL-10 rose again, particularly in the 2 µg/mL group, while the 3 µg/mL group declined. By day 21, IL-10 remained elevated in the 2 µg/mL and 3 µg/mL groups compared with control and the 1 µg/mL group. Data are presented as mean ± standard deviation (SD). Statistical significance between groups was determined using one-way ANOVA followed by a post-hoc multiple comparison test. Statistical comparisons between time points are indicated as follows: ns, not significant; *$p < 0.05$; **$p < 0.01$; ***$p < 0.001$; ****$p < 0.0001$. Analyses were performed using GraphPad Prism version 10.
